# Electroacupuncture Alleviated Referral Hindpaw Hyperalgesia via Suppressing Spinal Long-Term Potentiation (LTP) in TNBS-Induced Colitis Rats

**DOI:** 10.1155/2019/2098083

**Published:** 2019-03-11

**Authors:** Pei-Ran Lv, Yang-Shuai Su, Wei He, Xiao-Yu Wang, Hong Shi, Xiao-Ning Zhang, Bing Zhu, Yu Kan, Li-Zhen Chen, Qiao-Feng Wu, Shu-Guang Yu, Xiang-Hong Jing

**Affiliations:** ^1^Institute of Acupuncture and Moxibustion, China Academy of Chinese Medical Sciences, Beijing 100700, China; ^2^Chengdu University of Traditional Chinese Medicine, Chengdu 611137, China

## Abstract

Although referred pain or hypersensitivity has been repeatedly reported in irritable bowel syndrome (IBS) patients and experimental colitis rodents, little is known about the neural mechanisms. Spinal long-term potentiation (LTP) of nociceptive synaptic transmission plays a critical role in the development of somatic hyperalgesia in chronic pain conditions. Herein, we sought to determine whether spinal LTP contributes to the referral hyperalgesia in colitis rats and particularly whether electroacupuncture (EA) is effective to alleviate somatic hyperalgesia via suppressing spinal LTP. Rats in the colitis group (induced by colonic infusion of 2,4,6-trinitrobenzenesulfonic acid, TNBS), instead of the control and vehicle groups, displayed evident focal inflammatory destruction of the distal colon accompanied not only with the sensitized visceromotor response (VMR) to noxious colorectal distension (CRD) but also with referral hindpaw hyperalgesia indicated by reduced mechanical and thermal withdrawal latencies. EA at Zusanli (ST36) and Shangjuxu (ST37) attenuated the severity of colonic inflammation, as well as the visceral hypersensitivity and referral hindpaw hyperalgesia in colitis rats. Intriguingly, the threshold of C-fiber-evoked field potentials (CFEFP) was significantly reduced and the spinal LTP was exaggerated in the colitis group, both of which were restored by EA treatment. Taken together, visceral hypersensitivity and referral hindpaw hyperalgesia coexist in TNBS-induced colitis rats, which might be attributed to the enhanced LTP of nociceptive synaptic transmission in the spinal dorsal horn. EA at ST36 and ST37 could relieve visceral hypersensitivity and, in particular, attenuate referral hindpaw hyperalgesia by suppressing the enhanced spinal LTP.

## 1. Introduction

Visceral sensory inputs triggered by inflammation, psychological or environmental stress, and postinjury often produce vague, diffuse body sensations, and especially the referred pain at somatic targets [1–3]. It has been demonstrated that transient colonic inflammation leads to chronic visceral and hindpaw hypersensitivity in a subset of rats [4], and patients with IBS exhibit visceral hyperalgesia and cutaneous allodynia/hyperalgesia distributed in their lower extremities [5]. Although the convergence of somatic and visceral sensory circuits in the peripheral and central nervous systems (CNS) is deemed as the neuroanatomical evidence for the referral somatic hyperalgesia [6, 7], the functional neural basis of this phenomenon still remains elusive.

The spinal dorsal horn is the first relay site in the CNS that receives, integrates, and transmits periphery sensory information into higher regions of the brain. Visceral and somatic afferents converge into the same or adjacent spinal cord segments and synapse with intrinsic neurons in the spinal dorsal horn [8, 9]. Although the spinal long-term potentiation (LTP) of nociceptive synaptic transmission plays critical roles in the development and maintenance of hyperalgesia under somatic pathological pain conditions [10–12], its contribution to referral somatic hypersensitivity that resulted from inflammatory bowel diseases is still unclear.

Acupuncture is effective to alleviate gastrointestinal diseases [13–15] and has notable analgesic effects on multiple somatic and visceral pain disorders by modulating sensory information in spinal and supraspinal neural circuits [16, 17]. It has been shown that EA attenuates “central sensitization” by inhibiting the induction spinal LTP of C-fiber-evoked field potentials (CFEFP) in spinal nerve ligation rats [18]. Hence, the aim of the present study is to determine whether EA could relieve visceral hypersensitivity and, in particular, the referral somatic hyperalgesia via suppressing spinal LTP in TNBS-induced colitis rats.

## 2. Materials and Methods

### 2.1. Ethical Statement

Animal care and experimental procedures used in the current study were approved by the Institute of Laboratory Animal Sciences, China Academy of Medical Sciences (experimental animal license number: SCXK (Jing) 2014-0013). The study was carried out adhering to guidelines provided by the National Institutes of Health for the Care and Use of Laboratory Animals, and all efforts were made to minimize the suffering of animals. This study has also obtained ethics committee approval from the Institute Animal Welfare and Use Committee of IAM-CACMS (No. 20160520).

### 2.2. Animals

In the present study, a total of 64 male adult Sprague-Dawley rats weighing 180~200 g were used and randomly divided into four groups: control group (*n* = 15), vehicle group (*n* = 16), colitis group (*n* = 17), and EA group (*n* = 16). Female rats were not used in this experiment to eliminate changes in perception sensitivity due to the estrus cycle. Rats were housed in pairs under constant room temperature and humidity with 12 h light-dark cycles and allowed to acclimate to the housing conditions for seven days prior to the experiment.

### 2.3. TNBS-Induced Experimental Colitis

Intracolonic administration of TNBS was used to produce chronic colitis model as previously described [4, 19]. Briefly, rats underwent fasting overnight with free access to water for 16 h before TNBS administration. Under 2% isoflurane anesthesia (0.5 L/min, R580s, RWD Life Science, China), a mixture of TNBS (100 mg/kg body weight) with 50% ethanol (volume ratio 2 : 1) was instilled into the distal colon lumen (6~7 cm proximal to the anus) by plastic feeding tube (FTP-18-75; Instech Laboratories Inc., Plymouth Meeting, PA, USA) via the rectum. After TNBS infusion, rats were kept in a vertical position for at least 5 min to avoid leakage of the instilled intracolonic solutions. Then rats were placed back into cages to recover and regain consciousness. An equivalent volume of sterilized saline or ethanol was administered into control rats or vehicle rats, respectively. All of the rats were monitored daily for changes in body weight, body condition, physical appearance, and behavior following TNBS administration.

### 2.4. Visceromotor Response (VMR)

VMR was evoked by repeated colorectal distention (CRD) in lightly anesthetized rats, as described previously [20]. Firstly, anesthesia was induced by 4% isoflurane and maintained with 2% isoflurane for surgical procedures. A flexible latex balloon (3 cm long, 1.5 cm max diameter) was lubricated and inserted into the distal colon lumen via the anus (the tip of the balloon was 1 cm from the anus), and two electrodes made of Teflon-coated platinum wires were inserted into the external abdominal oblique muscle. After surgery, the anesthesia level was decreased to 1% isoflurane for electromyography (EMG) recording. The balloon was inflated by phasic distension (40, 60, and 80 mmHg, each lasting for 20 s) with a 5 min interval. The EMG signals were amplified (×5000), filtered (30~1000 Hz) by NL900D (Neurolog, Digitimer, America), then transmitted into PowerLab 8/35 (AD Instruments, Australia) and analyzed off-line by LabChart 7.1 software. The EMG reflex responding to each CRD stimulus was repeated 3 times and the outcomes were averaged. After all behavior tests were finished, rats were euthanized and the distal colon was removed for histopathological test to confirm the development of colitis.

### 2.5. Measurement of Mechanical Pain Threshold

Mechanical pain threshold was measured by using electronic von Frey filaments (37450-Dynamic Plantar Aesthesiometer, Ugo Basile, Italy) with a cut-off set at 50 g. Rats were placed in the plastic cages on perforated metal platform and allowed to habituate the environment for 30 min before testing. The metal filament was driven perpendicularly by a microcomputer controller onto the plantar of the hindpaw. Brisk withdraw or paw flinching was considered as positive response. Bilateral hindpaw mechanical withdrawal latencies were tested for 4 times with 5 min intervals and average values were calculated.

### 2.6. Measurement of Thermal Pain Threshold

Thermal pain threshold was measured by the Plantar Analgesia Meter (IITC 390G Plantar test, IITC Life Science Inc., Woodland Hills, Canada), as described [21]. Each rat was placed in an individual plexiglass enclosure compartment on the glass surface and allowed to habituate the environment for 30 min before testing. Thermal stimulus was emitted by a movable radiant heat source under the glass surface and was focused on the plantar surface of the hindpaw. The active intensity was set as 50% while idle intensity was 10%; cut-off time was set as 25 s to prevent potential tissue damage from sustained heating. Bilateral hindpaw thermal withdrawal latencies were tested for 4 times with 5 min intervals and average values were calculated.

### 2.7. Induction of Spinal LTP of C-Fiber-Evoked Field Potentials (CFEFP)

After being anesthetized by urethane (1.5 g/kg, i.p.), a tracheal intubation was performed to allow mechanical ventilation during the subsequent electrophysiological recording. Laminectomy was performed at the vertebrae T13-L2 to expose the lumbar enlargement of the spinal cord (L4-L5 level) [22]; the dura covering lumbosacral spinal segments was carefully removed. The vertebral column from T12 to L4 was firmly fixed in the frame with two clamps (ST-7R-HT, NARISHIGE, Japan). A pair of bipolar silver hook electrode was placed under the sciatic nerve proximal to the trifurcation for electrical stimulation, and the distance from the stimulation site to the spinal recording site was about 11 cm. All exposed spinal segments, sciatic nerve, and tissues were covered with (37°C) paraffin oil. After surgery, the animal was paralyzed with gallamine triethiodide (0.2 g/kg, i.p.) and artificially ventilated with a ventilator (SAR-830/P, CWE Inc., USA). Body temperature was maintained at 36.5–37.5°C via a feedback-controlled heating pad.

The CFEFP were recorded at a depth of 300–500 *μ*m under the dorsal surface of the L4–L5 spinal cord with a parylene-coated tungsten microelectrode (impedance 3 MΩ, FHC, USA) driven by a one-axis motorized stereotaxic micromanipulator (DMA-1550, NARISHIGE, Japan). The test stimulation (TS) of a single square pulse (0.5 ms, delivered every 2 s) was delivered by an isolated pulse stimulator (Model 2100, A-M Systems, USA) and applied to the sciatic nerve to measure the threshold of evoking the field potentials. The intensity was increased gradually from 0 V to that evoke field potential to define the threshold of CFEFP as previously described [18]. Following this measurement, the amplitude of the field potential did not continue to increase till the stimulus increased to a certain intensity, and this intensity was defined as test stimulus. A bandwidth of 0.1–500 Hz was used to remove artifacts without altering the CFEFP. The signals were amplified, filtered by a microelectrode AC amplifier (Model 1800, A-M Systems, USA), and transmitted to DELL Workstation via CED micro 1401 for recording and off-line analysis using the Spike 2 software (Cambridge Electronic Design, Cambridge, Cambridge, UK).

LTP of the CFEFP was induced by high-intensity and high-frequency electrical stimulation of the sciatic nerve, as described by Liu et al. and Liu and Sandkühler et al. [23, 24]. Briefly, the test stimulation (12-25 V, 0.5 ms, 6 times, 5 min intervals) was applied to the sciatic nerve to evoke spinal field potentials as baseline control. The mean amplitude of the control field potentials was obtained from an average of 6 individual test potentials and normalized as 100%. Then conditional stimulation (CS, 2 times intensity of CFEFP threshold, 0.5 ms, 100 Hz, 400 pulses given in 4 trains of 1 s duration with 10 s intervals) was delivered to the sciatic nerve to induce LTP. After CS, the same TS was delivered to the sciatic nerve again to evoke field potentials and the recording was continued for 3 h at least. The amplitude of the field potentials after CS was normalized and expressed as the percentage of the baseline control value. LTP was defined as at least a 20% increase in amplitude of the synaptic response and maintained for a minimum of 20 min following a brief high-frequency stimulation [25].

### 2.8. Electroacupuncture Treatment

**Figure 1:**
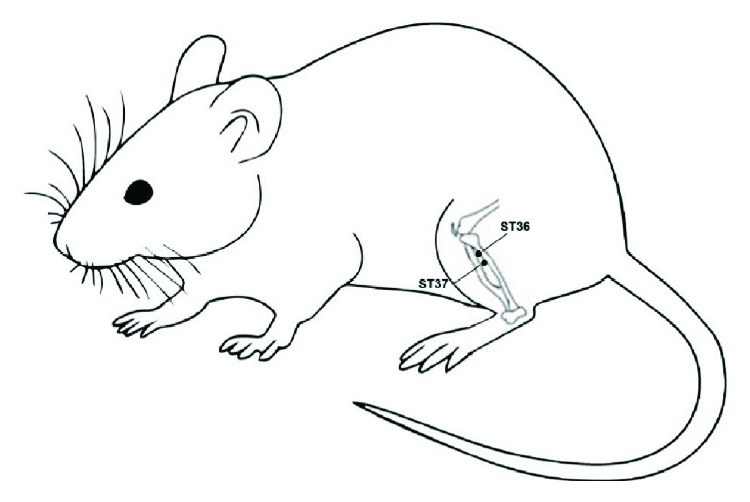
Schematic drawing of the location of acupoints ST36 and ST37 in a rat. ST36 is located at 5 mm lateral to the anterior tubercle of the tibia and 10 mm below the knee joint, and ST37 is located 5 mm below the ST36 and 1 mm lateral to the margin anterior tibiae.

Under 2% isoflurane anesthesia, EA treatment was applied at 24 hours after TNBS administration in rats. Acupoints ST36 (Zusanli) and ST37 (Shangjuxu) were selected in this study (Figure 1), which were widely used for alleviating various types of visceral pain and digestive system diseases in clinic [14, 26]. ST36 is located at 5 mm lateral to the anterior tubercle of the tibia and 10 mm below the knee joint [27]. ST37 is located at 5 mm below the ST36 and 1 mm lateral to the margin anterior tibiae in rats [28]. Electrical stimulation was delivered by Han's Acupoint Nerve Stimulator (HANS-100A, Nanjing Gensun Medical Technology Co. Ltd., China) via two pairs of stainless needles (0.18 mm diameter, 13 mm length; Beijing Zhongyan Taihe Medicine Co., Beijing, China) inserted into bilateral ST36 and ST37 (square waves; frequency: 2 Hz; intensity: 1 mA; duration: 30 minutes; depth: 3 mm) for 7 consecutive days.

### 2.9. Statistical Analysis

The area under the curve (AUC) for EMG activities during each 20 s of CRD was calculated using an in-house written computer program [27]. The VMR responding to each CRD was calculated by subtracting the EMG baseline value derived from the AUC of 20 s predistention period. The amplitude of the CFEFP after CS was normalized and expressed as the percentage of the control value. All data were expressed as mean ± standard error of the mean. Statistical analysis was conducted using GraphPad Prism 7 (GraphPad Prism Software Inc., San Diego, USA). Normality was checked for all analyses. Two-way ANOVA followed by Tukey's multiple comparison test was performed to evaluate EA's effects on VMR and hindpaw pain thresholds. Repeated measures of ANOVA followed by Newman-Keuls post hoc test were used for comparison between pre- and post-CS (for monitoring the LTP induction) or between the colitis and EA groups (for identifying EA's effect). The level of significance was set at *P* < 0.05.

## 3. Results

### 3.1. EA Attenuated Visceral Hypersensitivity in TNBS-Induced Colitis Rats

**Figure 2:**
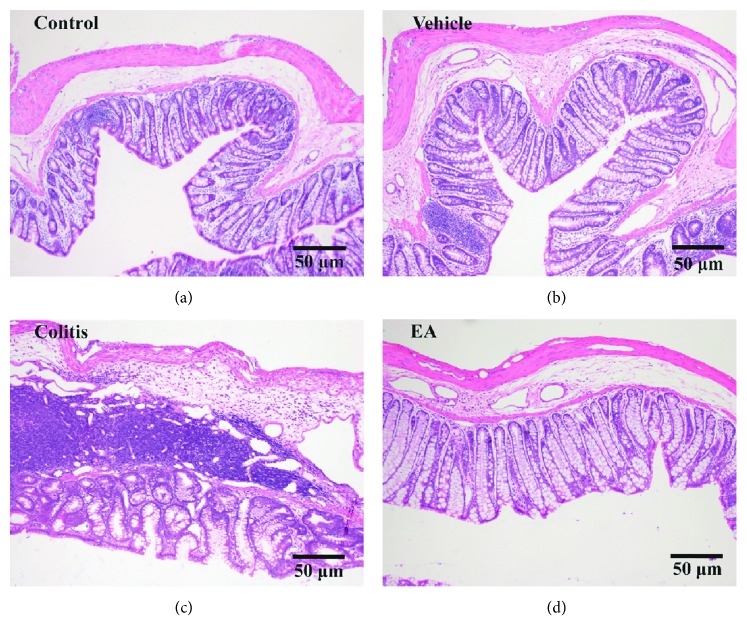
The distal colon was processed for H&E staining to evaluate TNBS-induced local damage. Colitis rats (c), instead of the control (a) and vehicle groups (b), exhibited severe colonic damage marked by massive transmural inflammatory cell infiltration and thickening of the colonic wall. However, rats in the EA group manifested slight architecture change of colon tissue and moderate transmural inflammatory cell infiltration (d).

The effect of EA on TNBS-induced colitis was confirmed by histological examination of colon tissues (5 cm proximal to the rectum). As compared with the control (Figure 2(a)) and vehicle groups (Figure 2(b)), colitis rats (Figure 2(c)) exhibited severe colonic damage marked by massive transmural inflammatory cell infiltration and thickening of the colonic wall. However, after 7 d EA treatment, rats manifested slight architecture change of colon tissue and limited transmural inflammatory cell infiltration (Figure 2(d)).

**Figure 3:**
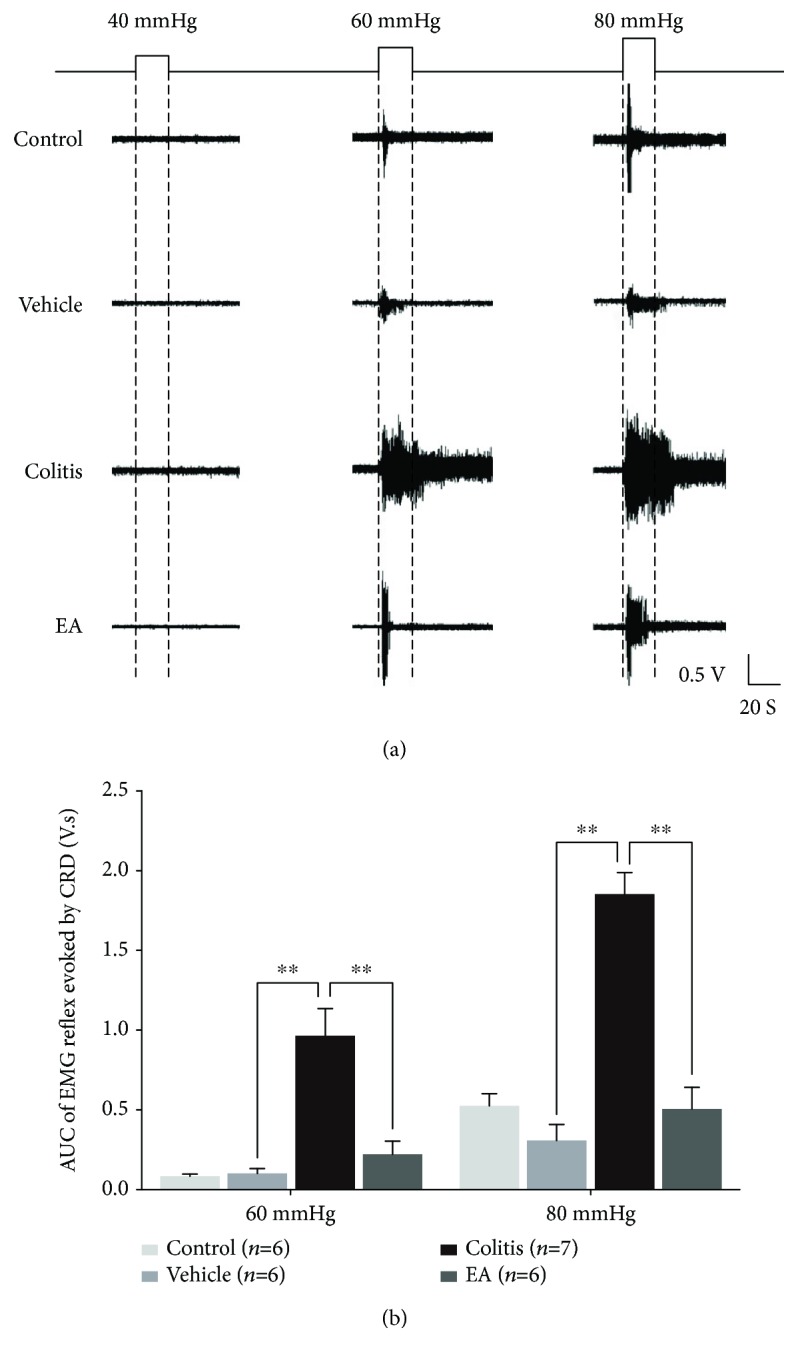
EA attenuated the enhanced VMR responding to noxious CRD (60 and 80 mmHg) in TNBS-induced colitis rats. Prominent VMR was evoked by noxious 60 and 80 mmHg CRD, instead of 40 mmHg CRD, in 1% isoflurane-anesthetized rats (a). The sensitized EMG reflexes evoked by noxious CRD in TNBS-treated rats were attenuated by EA at ST36 and ST37 (b). Data are expressed as mean ± SE (^∗∗^*P* < 0.01).

To determine the development of colonic mechanical hypersensitivity, the EMG reflex of external oblique abdominal muscle evoked by graded CRD (VMR) was collected 7 days after TNBS administration. Remarkable VMR was evoked by noxious 60 mmHg and 80 mmHg CRD, instead of 40 mmHg CRD, in 1% isoflurane-anesthetized rats. Of note, the EMG reflexes were parallel between the control (data not shown) and vehicle groups but were dramatically exaggerated in the colitis group (Figure 3(a)). More specifically, the area under the curve (AUC) value of EMG reflex in the colitis group was significantly higher than that in the vehicle group (colitis group vs. vehicle group: 60 mmHg: 0.965 ± 0.172 V.s vs. 0.100 ± 0.026 V.s, *P* < 0.01; 80 mmHg: 1.850 ± 0.132 V.s vs. 0.304 ± 0.099, *P* < 0.01, Figure 3(b)). In comparison with the colitis group, however, rats in the EA group showed a significant decrease in AUC following 60 mmHg and 80 mmHg CRD (EA group: 60 mmHg: 0.214 ± 0.083 V.s, *P* < 0.01; 80 mmHg: 0.507 ± 0.136 V.s, *P* < 0.01, Figure 3(b)). These data demonstrated that local inflammatory damage and visceral mechanical hypersensitivity in TNBS-induced colitis rats were attenuated by EA at ST36 and ST37, which were consistent with previous studies showing that EA intervention improved chronic visceral hypersensitivity produced by neonatal colonic injection of acetic acid [27] or chronic stress [29].

### 3.2. EA Alleviated Referral Hindpaw Hyperalgesia in TNBS-Induced Colitis Rats

Colitis rats exhibited abnormal posturing and behaviors, such as repeated licking of the lower abdomen, testicles, and hindpaws and a hunched posture, which started from several hours after TNBS administration. These abnormal behaviors were also described in other experimental visceral inflammatory models [30, 31].

**Figure 4:**
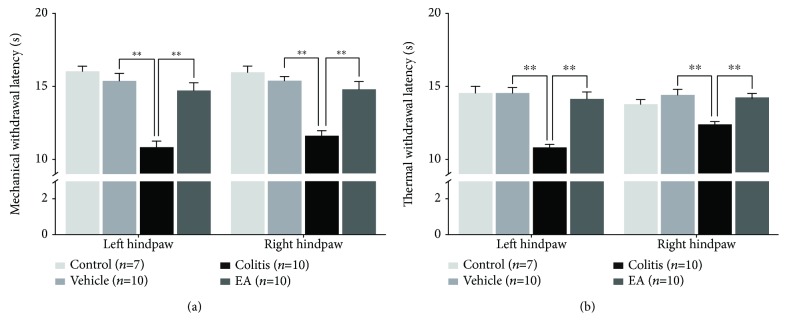
EA restored the referral hindpaw hyperalgesia in colitis rats. The mechanical (a) and thermal (b) withdrawal latencies of bilateral hindpaws in colitis rats were reduced in comparison with the vehicle group, indicating the development of referral hindpaw hypersensitivity after TNBS irritation, which could be alleviated by EA treatment. Data are expressed as mean ± SE (^∗∗^*P* < 0.01).

The hindpaw withdrawal latency to continuously increased von Frey stimulation was performed to evaluate referral somatic mechanical sensitivity. Rats in the vehicle group showed normal mechanical perception and had no significant difference with the control group (data not shown), whereas bilateral hindpaw withdrawal latencies were markedly decreased in colitis rats (colitis group vs. vehicle group: left: 10.858 ± 0.394 s vs. 15.380 ± 0.525 s, *P* < 0.01; right: 11.608 ± 0.370 s vs. 15.391 ± 0.289 s, *P* < 0.01, Figure 4(a)), indicating that TNBS colitis rats exhibited referral somatic mechanical hyperalgesia in the bilateral hindpaw. Nevertheless, the hindpaw withdraw latency of the rats in the EA group was increased significantly compared with that in the colitis group (EA group: left: 14.716 ± 0.532 s, *P* < 0.01; right: 14.819 ± 0.531 s, *P* < 0.01, Figure 4(a)).

The hindpaw withdrawal latency to thermal stimulation was tested by plantar test apparatus (Hargreaves method) to evaluate referral somatic thermal sensitivity. Similarly, rats in the vehicle group had normal somatic thermal perception. Bilateral hindpaw withdrawal latencies to thermal stimulation of the rats in the colitis group were deceased obviously compared with those in the vehicle group (colitis group vs. vehicle group: 10.849 ± 0.223 s vs. 14.588 ± 0.395 s, *P* < 0.01; right 12.441 ± 0.195 s vs. 14.499 ± 0.354 s, *P* < 0.01, Figure 4(b)), while EA treatment markedly alleviated the thermal hyperalgesia of bilateral hindpaws that resulted from intracolonic TNBS irritation (EA group: left: 14.213 ± 0.460 s, *P* < 0.01; right: 14.296 ± 0.257 s, *P* < 0.01, Figure 4(b)).

Taken together, bilateral referral hindpaw hyperalgesia was developed in TNBS-induced colitis rats, which has also been demonstrated in previous studies [4]. EA treatment was effective to alleviate both the visceral hypersensitivity and referral hindpaw hyperalgesia.

### 3.3. EA Inhibited the Facilitation of CFEFP in TNBS-Induced Colitis Rats

**Figure 5:**
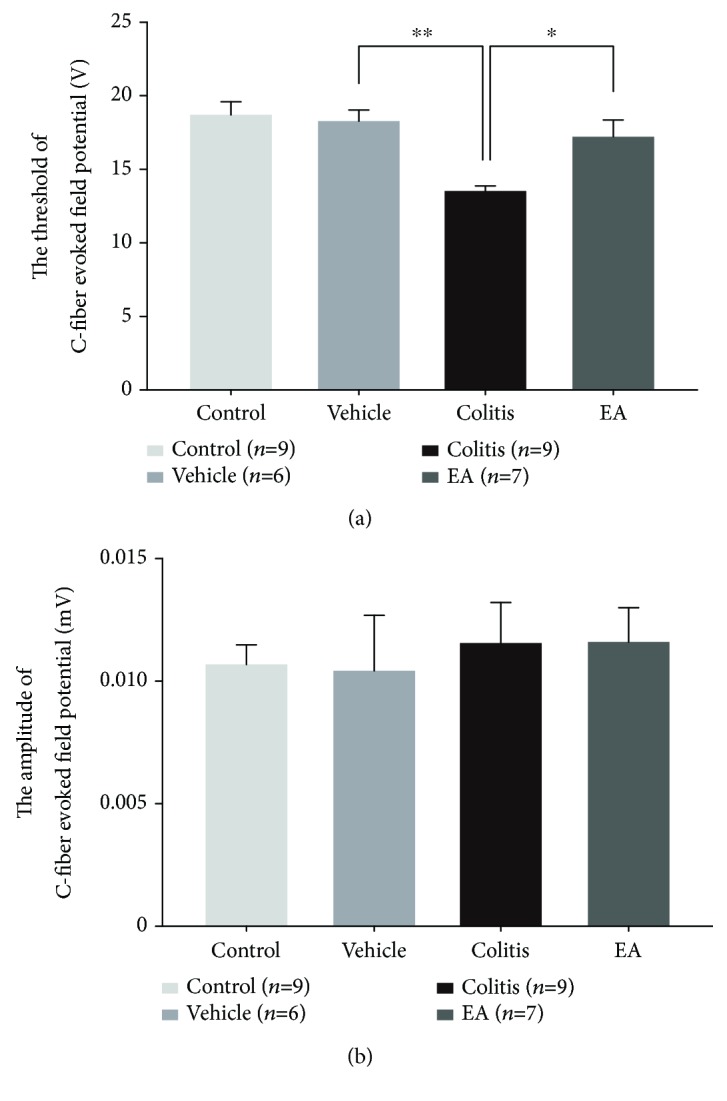
EA restored the downregulated threshold of C-fiber-evoked field potential (CFEFP) in colitis rats. The threshold for CFEFP induction was reduced in colitis rats, indicating the sensitization of somatic nociceptive transmission, which could also be attenuated by EA treatment (a). The amplitude of CFEFP showed no significant change among the four groups (b). Data was expressed as mean ± SE (^∗^*P* < 0.05, ^∗∗^*P* < 0.01).

To further explore whether the referral hindpaw hyperalgesia in TNBS-induced colitis rats resulted from hyperexcitability of nociceptive synaptic transmission in the spinal dorsal horn, the threshold and amplitude of CFEFP evoked by testing stimulation (TS) applied on the sciatic nerve (12-25 V, 0.5 ms, delivered every 5 min for 30 min) were examined in all groups of rats. Consistent with the behavioral outcomes, the threshold of CFEFP in the colitis group was significantly lower than that in the control (data not shown) and vehicle groups (colitis group vs. vehicle group: 13.56 ± 0.3379 V vs. 18.33 ± 0.7601 V, *P* < 0.01, Figure 5(a)), suggesting that the noxious C-component response in the spinal dorsal horn was facilitated in TNBS colitis rats. Moreover, EA inhibited the facilitation by markedly increasing the threshold of CFEFP (EA group: 17.29 ± 1.107, *P* < 0.05, Figure 5(a)). However, the amplitude of field potentials evoked by TS was not different among the four groups (Figure 5(b)).

### 3.4. EA Suppressed the Enhanced Spinal LTP in TNBS-Induced Colitis Rats

**Figure 6:**
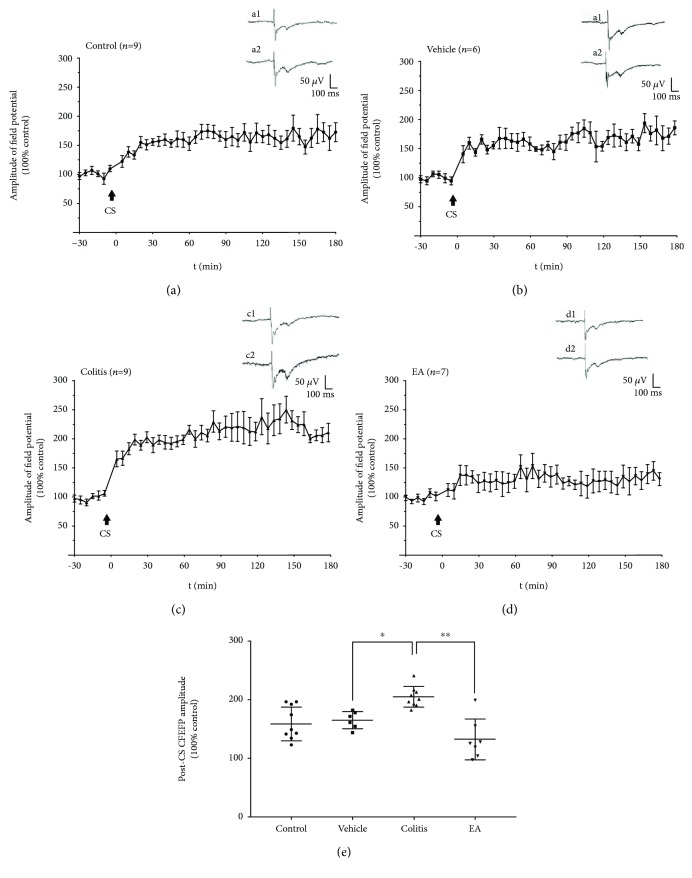
EA suppressed the facilitated spinal LTP in colitis rats. High-frequency and high-intensity conditioning stimulation (CS) was applied to the sciatic nerve to induce the spinal LTP at the L4-L5 segment in the control (a), vehicle (b), colitis (c), and EA (d) groups. After CS, the CFEFP was continuously recorded for at least 3 h. The amplitude of the field potentials after CS was normalized and expressed as the percentage of the baseline control value. Arrows indicate the time point of CS application. The enhanced spinal LTP in colitis rats was suppressed by EA treatment (e). Representative traces (a1, b1, c1, and d1) are the basal CFEFP recorded before CS; traces (a2, b2, c2, and d2) show the increased CFEFP obtained after CS. Data were expressed as mean ± SE (^∗^*P* < 0.05, ^∗∗^*P* < 0.01).

To address the plastic changes of spinal nociceptive synaptic transmission following visceral hypersensitivity, LTP was elicited by high-frequency and high-intensity electrical stimulation of the sciatic nerve and recorded in spinal lumbar enlargement segments. The conditional stimulation (CS, 2 times of the threshold of CFEFP) produced a prolonged increase in the amplitude of CFEFP which lasted for more than 3 h (Figures 6(a)–6(d)). The average amplitude in colitis rats was 202.70 ± 5.83% of the baseline control, which was significantly higher than those of the control (159.30 ± 9.48%) and vehicle groups (163.60 ± 5.98%, *P* < 0.05; Figure 6(e)). However, the average enhancement of LTP in the EA group (131 ± 13.12%) was significantly reduced (*P* < 0.01, Figure 6(e)). The electrophysiological recording results suggested that the efficacy of somatic nociceptive signaling transmission in the spinal dorsal horn was also exaggerated in colitis rats, which might lead to the development of the referral hindpaw hyperalgesia. EA treatment was likely to improve the referral somatic hypersensitivity in colitis rats via suppressing the facilitated spinal LTP.

## 4. Discussion

In the present study, we showed that TNBS-induced colitis rats exhibited visceral hypersensitivity and referral hindpaw hyperalgesia, both of which were attenuated by EA treatment at ST36 and ST37. In accordance with the behavioral phenotypes, threshold for evoking the CFEFP was lowered and spinal LTP was facilitated in colitis rats, suggesting that somatic nociceptive signaling transmission was exaggerated, which might serve as the functional neural substrate of the referral hindpaw hyperalgesia. Notably, EA restored the enhanced threshold of CFEFP and facilitated spinal LTP in colitis rats and suppressed the exaggerated nociceptive signaling transmission in the spinal dorsal horn.

Referral pain or hyperalgesia that arises from visceral disorders has been well documented and widely investigated [32–34]. Patients with IBS have both visceral and cutaneous hyperalgesia that is distributed mainly in lumbosacral dermatomes [5]. Previous studies demonstrated that noxious visceral stimulation induces expansion of the somatic convergent receptive field and sensitization of responses to mechanical stimuli [35]. Meanwhile, acute somatic noxious stimulus sensitizes CRD-responsive spinal neurons receiving viscerosomatic convergent inputs [36]. Ample evidence shows that the viscerosomatic convergence is a common phenomenon residing in the spinal dorsal horn. It was also found that a group of spinal Lamina I anterolateral projection neurons received monosynaptic convergence of afferent inputs from both somatic and visceral regions, indicating that Lamina I might be the first site in CNS for somato-visceral convergence processing. More importantly, another subgroup of Lamina I neurons received suprathreshold or subthreshold excitatory visceral inputs together with inhibitory somatic inputs, and the overall inhibition in these neurons can be caused by somatic A*δ* and C-afferents, instead of A*β*-afferents [37]. These findings suggested that somato-visceral convergence may not only contribute to referral pain but also be a neural basis for shutting down visceral nociceptive transmission by adequate somatic stimuli, such as electroacupuncture, manual acupuncture, transcutaneous electrical nerve stimulation (TENS), and percutaneous peripheral nerve stimulation. In fact, nociceptive visceral inputs could be inhibited by acupuncture applied to homotopic acupoint, in which the spinal dorsal horn plays an important role in proceeding and integrating the inhibitory outcomes [38].

Our previous studies showed that the sensitized area induced by acute colorectal mucosal injury was distributed in the same dermatomes as the colorectum, named homotopic acupoint. The C-fiber of ipsilateral sciatic nerve was more sensitive to the electrical stimulation in the sensitized somatic region [39]. Local nociceptive neuropeptides such as substance P (SP) and calcitonin gene-related peptide (CGRP) in the somatic hypersensitive area were highly expressed after visceral injury [40]. Furthermore, the noxious stimuli from the convergent cutaneous receptive field could inhibit the activity of visceral nociceptive neurons [41], indicating that the somatic hypersensitive area was not only a spot reflecting the visceral disorders but also a target for disease treatment. Consequently, the effect of the sensitized homotopic acupoint to both the visceral and referral somatic hyperalgesia should be confirmed to elucidate the significance of the sensitized acupoints. The present study demonstrated that EA at ST36 and ST37, which are the perisegmental acupoints as the colorectum innervation, was effective to relieve both the visceral hypersensitivity and referral somatic hyperalgesia. Our result is consistent with a report that EA at ST36, but not at BL43 (heterosegmental acupoint), significantly suppressed the visceral motor responses to CRD [27]. Moreover, it is important to emphasize that the present study examined visceral and referral somatic hypersensitivity at 7 days after TNBS administration, while the efficacy of EA was achieved after 7 consecutive days of application, which indicated that EA at the perisegmental acupoint had accumulating therapeutic effects on both visceral and referral somatic hypersensitivity in colitis rats. Additionally, our data demonstrated that the referral somatic region with sensitized perception is not only a symptom of chronic visceral pain disorders but also an optimal location of needling for treatment.

Although the neuroanatomical evidence of the convergence of somatic and visceral inputs has been well established [36, 41], the functional neural basis for referred pain in the spinal cord level is still unclear. The LTP of CFEFP is considered as a fundamental mechanism of “central sensitization” in the spinal dorsal horn under pathological pain condition [42]. Previous studies demonstrated that somato-visceral sensitization is independent of supraspinal neural circuits, while ionotropic glutamate receptors in the spinal cord are involved in the sensitization [36]. EA attenuated diverse visceral pain disorders via multiple neurobiological pathways [43, 44]. Although the specific aspects where EA modifies nociceptive transmission are still not clearly identified, EA has been proven to activate the ascending sensory pathways such as the spinal dorsal horn and the thalamus or the descending pain inhibitory pathways [17]. Our study demonstrated that EA modulated the plasticity of nociceptive synaptic transmission in the spinal dorsal horn so as to relieve the referral somatic hyperalgesia under visceral hypersensitivity condition.

Several potential neurotransmitters could be involved in the sensitization of spinal LTP. It has been shown that the N-methyl-D-aspartic acid receptors (NMDARs) play an important role in the development of hyperalgesia, and the sensitization of spinal pain projection neurons is attenuated by the NMDAR antagonist [45]. Colonic inflammation persistently increased the expression of NMDAR in the spinal dorsal horn [46, 47], which may contribute to both visceral and referral somatic hypersensitivity in a subset of rats. The administration of dextromethorphan, a NMDA antagonist, could alleviate the visceral and referred somatic hypersensitivity of IBS patients [46, 48]. EA can also affect the progress of experimental inflammatory pain by modulating the expression of NMDARs in primary sensory neurons [49]. The role of NMDAR in EA-relieved visceral and referral somatic hypersensitivity needs to be further explored. In addition, the endogenous opioid system is a well-accepted hypothesis for EA's analgesic effects [50, 51].

In conclusion, the present study demonstrated that visceral hypersensitivity and referral hindpaw hyperalgesia coexist in TNBS-induced colitis rats, which might be attributed to the enhanced spinal LTP of nociceptive synaptic transmission. EA at ST36 and ST37 could relieve visceral hypersensitivity and, in particular, attenuate referral hindpaw hyperalgesia by suppressing the enhanced spinal LTP.

## Data Availability

The data used to support the findings of this study are available from the corresponding author upon request.

## References

[1] Mayer E. A., Raybould H. E. (1990). Role of visceral afferent mechanisms in functional bowel disorders. *Gastroenterology*.

[2] Mayer E. A., Gebhart G. F. (1994). Basic and clinical aspects of visceral hyperalgesia. *Gastroenterology*.

[3] Gebhart G. F. (2000). IV. Visceral afferent contributions to the pathobiology of visceral pain. *American Journal of Physiology-Gastrointestinal and Liver Physiology*.

[4] Zhou Q., Price D. D., Caudle R. M., Verne N. G. (2008). Visceral and somatic hypersensitivity in a subset of rats following TNBS-induced colitis. *Pain*.

[5] Verne N. G., Robinson M. E., Price D. D. (2001). Hypersensitivity to visceral and cutaneous pain in the irritable bowel syndrome. *Pain*.

[6] Pierau F.-K., Fellmer G., Taylor D. C. M. (1984). Somato-visceral convergence in cat dorsal root ganglion neurones demonstrated by double-labelling with fluorescent tracers. *Brain Research*.

[7] Farrell K. E., Keely S., Graham B. A., Callister R., Callister R. J. (2014). A systematic review of the evidence for central nervous system plasticity in animal models of inflammatory-mediated gastrointestinal pain. *Inflammatory Bowel Diseases*.

[8] D'Mello R., Dickenson A. H. (2008). Spinal cord mechanisms of pain. *British Journal of Anaesthesia*.

[9] Cordero-Erausquin M., Inquimbert P., Schlichter R., Hugel S. (2016). Neuronal networks and nociceptive processing in the dorsal horn of the spinal cord. *Neuroscience*.

[10] Ikeda H., Kiritoshi T., Murase K. (2009). Synaptic plasticity in the spinal dorsal horn. *Neuroscience Research*.

[11] Luo C., Kuner T., Kuner R. (2014). Synaptic plasticity in pathological pain. *Trends in Neurosciences*.

[12] Ruscheweyh R., Wilder-Smith O., Drdla R., Liu X. G., Sandkühler J. (2011). Long-term potentiation in spinal nociceptive pathways as a novel target for pain therapy. *Molecular Pain*.

[13] Okada K., Kawakita K. (2009). Analgesic action of acupuncture and moxibustion: a review of unique approaches in Japan. *Evidence-based Complementary and Alternative Medicine*.

[14] Ma X. P., Hong J., An C. P. (2014). Acupuncture-moxibustion in treating irritable bowel syndrome: how does it work?. *World Journal of Gastroenterology*.

[15] Langhorst J., Wulfert H., Lauche R. (2015). Systematic review of complementary and alternative medicine treatments in inflammatory bowel diseases. *Journal of Crohn’s and Colitis*.

[16] Zhao J.-j., Rong P.-j., Shi L., Ben H., Zhu B. (2016). Somato stimulation and acupuncture therapy. *Chinese Journal of Integrative Medicine*.

[17] Zhao Z.-Q. (2008). Neural mechanism underlying acupuncture analgesia. *Progress in Neurobiology*.

[18] Xing G.-G., Liu F.-Y., Qu X.-X., Han J.-S., Wan Y. (2007). Long-term synaptic plasticity in the spinal dorsal horn and its modulation by electroacupuncture in rats with neuropathic pain. *Experimental Neurology*.

[19] Morris G. P., Beck P. L., Herridge M. S., Depew W. T., Szewczuk M. R., Wallace J. L. (1989). Hapten-induced model of chronic inflammation and ulceration in the rat colon. *Gastroenterology*.

[20] Palecek J., Willis D. W. (2003). The dorsal column pathway facilitates visceromotor responses to colorectal distention after colon inflammation in rats. *Pain*.

[21] Hargreaves K., Dubner R., Brown F., Flores C., Joris J. (1988). A new and sensitive method for measuring thermal nociception in cutaneous hyperalgesia. *Pain*.

[22] Lien G. F. (2005). *Induction of Long-Term Potentiation in Dorsal Horn Neurons and Expression of Immediate Early Genes Zif and Arc [M.S. Thesis]*.

[23] Liu X. -G., Morton C. R., Azkue J. J., Zimmermann M., Sandkühler J. (1998). Long-term depression of C-fibre-evoked spinal field potentials by stimulation of primary afferent A*δ*-fibres in the adult rat. *European Journal of Neuroscience*.

[24] Liu X.-G., Sandkühler J. (1995). Long-term potentiation of C-fiber-evoked potentials in the rat spinal dorsal horn is prevented by spinal N-methyl-D-aspartic acid receptor blockage. *Neuroscience Letters*.

[25] Liu X.-G., Sandkühler J. (1997). Characterization of long-term potentiation of C-fiber–evoked potentials in spinal dorsal horn of adult rat: essential role of NK1 and NK2 receptors. *Journal of Neurophysiology*.

[26] Li H., He T., Xu Q. (2015). Acupuncture and regulation of gastrointestinal function. *World Journal of Gastroenterology*.

[27] Xu G.-Y., Winston J. H., Chen J. D. Z. (2009). Electroacupuncture attenuates visceral hyperalgesia and inhibits the enhanced excitability of colon specific sensory neurons in a rat model of irritable bowel syndrome. *Neurogastroenterology & Motility*.

[28] YAN J., ZHANG H., CHEN C.-t., YANG Q.-y., LIAO W.-f., CHEN P.-g. (2009). Effects of electroacupuncture at Shangjuxu (ST 37) on interleukin-1*β* and interleukin-4 in the ulcerative colitis model rats. *Journal of Traditional Chinese Medicine*.

[29] Zhou Y. Y., Wanner N. J., Xiao Y. (2012). Electroacupuncture alleviates stress-induced visceral hypersensitivity through an opioid system in rats. *World Journal of Gastroenterology*.

[30] Wesselmann U., Czakanski P. P., Affaitati G., Giamberardino M. A. (1998). Uterine inflammation as a noxious visceral stimulus: behavioral characterization in the rat. *Neuroscience Letters*.

[31] Ness T. J., Metcalf A. M., Gebhart G. F. (1990). A psychophysiological study in humans using phasic colonic distension as a noxious visceral stimulus. *Pain*.

[32] Hockley J. R. F., González-Cano R., McMurray S. (2017). Visceral and somatic pain modalities reveal NaV1. 7-independent visceral nociceptive pathways. *The Journal of Physiology*.

[33] Gebhart G. F., Ness T. J. (1991). Central mechanisms of visceral pain. *Canadian Journal of Physiology and Pharmacology*.

[34] Head H. (1893). On disturbances of sensation with especial reference to the pain of visceral disease. *Brain*.

[35] Euchner-Wamser I., Sengupta J. N., Gebhart G. F., Meller S. T. (1993). Characterization of responses of T2-T4 spinal cord neurons to esophageal distension in the rat. *Journal of Neurophysiology*.

[36] Peles S., Miranda A., Shaker R., Sengupta J. N. (2004). Acute nociceptive somatic stimulus sensitizes neurones in the spinal cord to colonic distension in the rat. *The Journal of Physiology*.

[37] Luz L. L., Fernandes E. C., Sivado M., Kokai E., Szucs P., Safronov B. V. (2015). Monosynaptic convergence of somatic and visceral C-fiber afferents on projection and local circuit neurons in lamina I: a substrate for referred pain. *Pain*.

[38] Rong P.-J., Zhu B., Huang Q. F., Gao X. Y., Ben H., Li Y. H. (2005). Acupuncture inhibition on neuronal activity of spinal dorsal horn induced by noxious colorectal distention in rat. *World Journal of Gastroenterology*.

[39] Xu J., Wu Q., Lin R. (2015). Electrophysiological characteristics of sensitized acupoints after acute intestinal mucosal injury in rats. *Zhen Ci Yan Jiu= Acupuncture Research*.

[40] He W., Wang X.-Y., Shi H. (2017). Cutaneous neurogenic inflammation in the sensitized acupoints induced by gastric mucosal injury in rats. *BMC Complementary and Alternative Medicine*.

[41] Ness T. J., Gebhart G. F. (1991). Interactions between visceral and cutaneous nociception in the rat. I. Noxious cutaneous stimuli inhibit visceral nociceptive neurons and reflexes. *Journal of Neurophysiology*.

[42] Rygh L. J., Svendsen F., Fiskå A., Haugan F., Hole K., Tjølsen A. (2005). Long-term potentiation in spinal nociceptive systems—how acute pain may become chronic. *Psychoneuroendocrinology*.

[43] Sun S., Cao H., Han M., Li T. T., Zhao Z. Q., Zhang Y. Q. (2008). Evidence for suppression of electroacupuncture on spinal glial activation and behavioral hypersensitivity in a rat model of monoarthritis. *Brain Research Bulletin*.

[44] Kim H. N., Park J. H., Kim S. K. (2008). Electroacupuncture potentiates the antiallodynic effect of intrathecal neostigmine in a rat model of neuropathic pain. *The Journal of Physiological Sciences*.

[45] Miranda A., Mickle A., Bruckert M., Kannampalli P., Banerjee B., Sengupta J. N. (2014). NMDA receptor mediates chronic visceral pain induced by neonatal noxious somatic stimulation. *European Journal of Pharmacology*.

[46] Zhou Q., Price D. D., Caudle R. M., Verne G. N. (2009). Spinal NMDA NR1 subunit expression following transient TNBS colitis. *Brain Research*.

[47] Liu M., Kay J. C., Shen S., Qiao L.-Y. (2015). Endogenous BDNF augments NMDA receptor phosphorylation in the spinal cord via PLC*γ*, PKC, and PI3K/Akt pathways during colitis. *Journal of Neuroinflammation*.

[48] Verne G. N., Price D. D., Callam C. S., Zhang B., Peck J., Zhou Q. (2012). Viscerosomatic facilitation in a subset of IBS patients, an effect mediated by N-methyl-D-aspartate receptors. *The Journal of Pain*.

[49] Wang L., Zhang Y., Dai J., Yang J., Gang S. (2006). Electroacupuncture (EA) modulates the expression of NMDA receptors in primary sensory neurons in relation to hyperalgesia in rats. *Brain Research*.

[50] Han J. S., Terenius L. (1982). Neurochemical basis of acupuncture analgesia. *Annual Review of Pharmacology and Toxicology*.

[51] Han J.-S. (2004). Acupuncture and endorphins. *Neuroscience Letters*.

